# Informing and Sustaining Participation of Lived Experience in the Suicide Prevention Workforce

**DOI:** 10.3390/ijerph20043092

**Published:** 2023-02-10

**Authors:** Jacinta Hawgood, Jurgita Rimkeviciene, Mandy Gibson, Martina McGrath, Bronwen Edwards, Victoria Ross, Tracee Kresin, Kairi Kolves

**Affiliations:** 1 Australian Institute for Suicide Research and Prevention, World Health Organization Collaborating Centre for Research and Training in Suicide Prevention, School of Applied Psychology, Griffith University, Brisbane, QLD 4122, Australia; 2 Suicide Research Center, Institute of Psychology, Faculty of Philosophy, Vilnius University, 03100 Vilnius, Lithuania; 3 Roses in the Ocean, Brisbane, QLD 4006, Australia

**Keywords:** lived experience, suicide prevention, workforce, peer work, workplace support

## Abstract

Background: Currently, there is no comprehensive study focused on identifying what is needed to support ongoing participation within the suicide prevention lived experience workforce (LEW). It is unclear what specific factors may impede or support ongoing participation in the LEW. The aim of this study was to explore the experiences of suicide prevention LEW in terms of its sustainability. Method: A qualitative interview method was utilised, with a purposive sample of participants who had engaged in the LEW for at least 12 months. The sample comprised 13 individuals (nine females, four males) who engaged in multiple LEW roles, with over half (54%) working in the LEW for more than 5 years. Data were analysed using thematic analysis. Results: Five main themes were identified: support, passion, personal impact, training, and work diversity. Each theme offers perspectives about the challenges participants face within the suicide prevention LEW. Conclusion: Challenges faced are both similar to those found in the broader MH sector and unique to suicide prevention. Findings suggest that managing expectations of the LEW is important and can inform the creation of guidelines for a supported and sustainable suicide prevention LEW.

## 1. Introduction

The multiple workforce roles of people with a lived experience of suicide, i.e., those who have experienced suicidal thoughts, survived a suicide attempt, cared for someone through suicidal crisis, or been bereaved by suicide [1], are now acknowledged as essential to the fabric of suicide prevention both in Australia and internationally [2,3,4]. Those with lived experience of suicide may contribute to suicide prevention efforts through participation in tasks such as project codesign, development, implementation, and evaluation of programs, policy advising, awareness-raising speaker engagements, undertaking or collaborating in research, and supporting others who are suicidal or bereaved by suicide through peer work [3,5].

As reported in different national guidelines for involvement of those with lived experience into the general mental health workforce [6,7], there are several principles and values important to mental health lived experience workforce (LEW) that are relevant also to the suicide prevention LEW. These range from values of equality, self-determination, and reciprocity to important points of clarity around consumer versus carer skills and worker-role identities and contexts [6]. However, the suicide-specific focus and experience makes suicide prevention LEW distinct from the broader mental health LEW. Suicidality and mental health issues do overlap, and the contribution of mental illness to suicide cannot be denied [8]. However, not all those who suicide have a mental illness, and, more importantly, not all those who have a mental illness are suicidal [9,10]. Notably, given that suicide is a behaviour and not a mental illness [11], the direct provision of support to those in suicidal distress may be very different to the support required for mental health experiences. Existing guidelines for the training and support of mental health LEW [12] lack tailored components deemed essential to the training and support of the suicide prevention LEW, such as their ability to discuss suicide safely without increasing suicide risk or stigma, as well as their own safety in terms of suicide risk [13]. Furthermore, the skills required by the suicide prevention LEW for supporting those in suicidal distress through to those bereaved by suicide demand a nuanced understanding of impacts of suicide-related stigma and the shame, guilt, and other internalised negative experiences associated with suicide lived experience [14,15]. Specialised skills relevant to suicide prevention are acknowledged in the National Lived Experience (Peer) Workforce Development Guidelines as being challenging to implement [6]. Furthermore, the majority of existing mental health LEW is in mental health and clinical service delivery contexts, where people experiencing suicidal distress may not frequently present or indeed meet eligibility criteria for care due to the severity of their crisis [16]. The move more recently to support and respond to people experiencing suicidality within community-based settings, which are nonclinical in nature and which are traditionally less resourced (in terms of funds and human resources), necessitates suicide-specific workforce support [17]. Different recruitment, skill development, and support mechanisms are required for supporting the rapid growth of nonclinical care and support options in the suicide prevention sector [14].

There is a small body of emerging research that has explored what motivates a person to become involved in the suicide prevention LEW [18,19,20], the potential benefits of participation [3,21], and the challenges involved in having a suicide prevention LEW [22,23]. However, we were unable to identify any guidelines for supporting their initial and sustained engagement. Sustainability of the mental health or suicide prevention workforce is of major importance in cultivating future workforce capabilities and the overall infrastructure of these sectors [24]. Research has demonstrated the negative impacts on members of the mental health LE workforce [25], yet we were unable to find studies on impacts specific to suicide prevention LEW. However, significant research has been conducted on the impacts of working in suicide prevention generally (mostly impacts on the clinical workforce) due to specific experiences of losing a client to suicide and/or having a client attempt suicide [26,27], with findings highlighting the early departure from this specific work of those impacted [28]. Some research into the suicide prevention LEW has already highlighted the importance of meeting ongoing care needs, including formalised support peer networks and post-training support [20]. Other research has highlighted the positive impacts of suicide prevention lived experience training, including significant gains in knowledge, self-efficacy, and confidence [29]. However, to our knowledge, there is no purposeful and comprehensive study focused on identifying what is needed to support ongoing participation within the suicide prevention LEW.

Enhancing capabilities and workforce systems and responses to support their members who themselves have a lived experience of suicide, however, is not yet fully understood with regard to sustainability of this workforce [20]. It would seem prudent, therefore, to understand what is required to develop related policies and guidelines to support ongoing contributions of those in the suicide prevention LEW before its further expansion. This would provide clarity in the sector as this workforce grows.

Therefore, the aim of this study was to explore the experiences of suicide prevention LEW to identify the issues that are critical to continued participation in the suicide prevention LEW.

## 2. Materials and Methods

### 2.1. Participants

A purposive sampling strategy was applied to the study to ensure a range of participants with lived experience of suicide who had engaged in the LEW for at least 12 months. This timeframe was chosen in order to obtain perspectives reflective of a wider range and duration of experience in the workforce in terms of facing different challenges and working through them in order to be able to reflect on the sustainability issues. The sample comprised 13 individuals (nine females, four males), with ages ranging from 26 to 78 years (*M* = 52.1, *SD* = 17.3). The participants’ self-described lived experience was experience of their own suicidality (*n* = 8, 62%), being bereaved by suicide (*n* = 6, 46%), and being a carer for someone who has been or is suicidal *(n* = 5, 38%). Four of these participants (31%) described having more than one of these experiences (two had all three types of experiences; two had been suicidal themselves and were a carer or bereaved by suicide). As shown in Table 1, participants had involvement across a broad range of activities within the LEW. The most common types of LEW activities that participants had been involved with were those related to providing lived experience advice or guidance, including as part of reference, working or advisory groups (84.6%), codesigning programs or services (76.9%), and strategic planning for suicide prevention organisations (53.8%). Over two-thirds (69.2%) of participants had shared their stories as a lived experience speaker. The majority (54%) had actively worked in the lived experience sector for more than 5 years.

### 2.2. Recruitment and Procedure

Individuals who completed either Our Voice in Action or Voices of In-Sight trainings, hosted by Roses in the Ocean [30], were contacted. These two programs are the only training programs in Australia for people with lived experience of suicide, and they are attended by participants of LEW from different organisations and communities across the country. Therefore, this national reach allowed capturing a wide range of experiences in the LEW. Three rounds of letters of invitation to participate were sent to all individuals who completed the between March 2018 and August 2019 across Australia, inviting all participants who had been in the LEW for at least a year, and contacting of participants was conducted in parallel to data collection and analysis. Eleven individuals who agreed to participate in the interviews were recruited this way. Given that the study was concerned with the sustainability of the workforce, of relevance were also perspectives of those leaving the LEW. One participant who had stepped down from LEW was identified in initial recruitment, and a snowballing technique was used to identify an additional two participants who discontinued their participation after more than 1 year of involvement (irrespective of their completion of training). Interviews were conducted from August 2020 to March 2021. The principle of data saturation was applied so that sampling was ceased when no new themes were identified in the interview data, including data from the two interviews with participants leaving the workforce recruited additionally [31].

For participant safety, interviews were undertaken by a female registered psychologist (M.G.), who did not have prior relationship with the participants, who is not a member of RITO organisation, and who has specialist experience in assessment and response for people experiencing suicidal crises. Due to COVID-19 restrictions, and to allow facilitation across Australia, all interviews were conducted over the phone. An information package about the study, together with consent forms, was emailed to the participants prior to the interview. The interviews started with a brief explanation of the purpose of the study, and participants were reminded they could discontinue the interview at any time should they become distressed or uncomfortable. A semi-structured interview was specifically developed for this purpose. The questions focused both on the experiences of the participants in the workforce (e.g., Have you had any difficulties while engaging in this work? If yes, can you describe these and how you got through them?) and on the sustainability of the LEW, both directly (e.g., “In terms of sustaining the lived experience workforce, what would you say is the greatest area of need?” “What strategies do you currently use to keep yourself safe and well participating in the LEW?”) and indirectly (e.g., “If you felt that continuing in the lived experience workforce was too much, how would you navigate this?”). Questions about the experiences and perceptions of RITO training and reasons for living were also included in the interview, but these were not analysed in the present study when the responses did not raise issues related to LEW sustainability. The average length of the interviews was 45–60 min. All interviews were audio-recorded and, in preparation for data analysis, transcribed verbatim by a company providing transcription services.

Ethical clearance was obtained for this study from the Griffith University Human Research Ethics Committee (GU HREC 2018/315). The article followed COREQ guidelines for reporting qualitative studies [32].

### 2.3. Analysis

Thematic analysis (TA) was chosen to analyse the data, following Braun and Clarke’s [33] six-step guide, because using this approach aims to arrive at identifying patterns of shared meaning, which was in line with our research questions; moreover, this method is theoretically flexible [34] and adaptive to a critical realism position we hold [35]. We used an inductive approach, where the themes were directed by the content of the data [33] and considered semantic rather than latent themes [33] to directly capture the participants’ voiced reflection about LEW sustainability. Initial data coding was undertaken by J.R., who had no direct experience with the LEW, to minimise impact of own prior direct LEW experience to analysis. M.M., who has 8 years of experience working within the LEW in different roles, and J.H., who has extensive experience evaluating LEW training and also some training of those in LEW, provided consistent input into the emerging thematic structure. J.R., J.H., M.G., and M.M. engaged in group discussions, during which separate codes were reviewed, before being merged into themes and subthemes, and the final codebook was created. Group discussions on the coding were used to increase the dependability of findings [36]. Differences in views in these meetings were used to clarify the assumptions each author had, to see more clearly the competing viewpoints from which the participants’ statements could be understood, and to arrive at a mutually acceptable decision about the final coding framework that could capture these competing interpretations. B.E., who has 12 years of experience within the LEW, reviewed the codebook and provided feedback on the final thematic structure, mainly in terms of labelling of themes/subthemes and organisation of subthemes into themes. This allowed capturing of the language most prevalently shared within the current LEW framework, for the results to be most useful for the members of the LEW. We used the 15-point checklist for good thematic analysis by Braun and Clarke [37] to review the analysis process and ensure its rigor.

The NVivo 12 software package (QSR International, Melbourne, Australia) was used to manage the analysis.

## 3. Results

Five main themes were identified within the data: support, passion, personal impact of LEW, training, and work diversity within the LEW. Each theme, with subthemes, is described below with supporting quotes.

### 3.1. Support

All participants spoke about the need to receive ongoing outside support to be able to continue doing the lived experience work.

“*It’s incredibly challenging, demanding work. I mean I keep going back to rendering yourself vulnerable. That needs an enormous amount of support and, when that doesn’t happen, then it’s easy enough to go down the tube. So again, it gets back to checking in on people to make sure that, you know—having those specific connections that people can talk to you about how they’re feeling, how they’re travelling and to support that particular person in a way that that person needs to be supported*.”

The different types of support mentioned as needed were support within the peer network, system-level support, and workplace support. Participants were mindful of other types of support, less related to LEW, that they themselves used and listed as helpful. These were informal connections with, e.g., family and friends, as well as formal support from mental healthcare workers (clinician, psychologist, and GP).

Support within a peer network involves connecting with others who are doing similar work and have lived experience themselves. This can be in different forms varying from less to more formal: just catch-ups on the go, debriefing, regular group meetings, and peer-to-peer supervision. Participants spoke that such peer support is indispensable and not comparable to other types, because it both gives a sense of direct support (such as having opportunities for sharing, hearing about good practices elsewhere), and sense of connectedness.

“*They’re the good things, and what keeps me going is talking to other people that have been through shit like me. Because you realise you’re not so alone. Other people have been through hell too. You can share stuff, validate, and help people get through that to know that they can come out the other end*.”

System-level support needed was described in terms of arrangements within the workplace setting necessary for the LEW. These include flexibility in work arrangements, being paid for work, and having work employee assistance programs (EAPs).

“*I think it is a little bit hard for me as far as that goes, just trying to still work 3 days a week, and then how do I get—if I wanted to not do that 3 days a week, then I need to be able to earn an income from these talks*.”

Direct workplace support describes the connections within the organisation necessary to feel able to continue the work. Participants wanted to have debriefing and supervision in the organisation (either with direct managers or colleagues), as well as feel that open and respectful communication is valued and heard, but not turned into mere risk management.

“*My issue with my workforce is, when I go to anyone, or the manager and say, hey, I just need 10 min, I’ve had a really hard situation, this is the situation, and usually I’m angry about something because the system has failed, or this person’s not getting the support they need because they don’t fit the criteria. So, when I’m passionately irritated about that, I always consistently get told that, maybe you’re not coping and maybe you need some sort of assistance*.”

Providing ongoing support, as opposed to a one-off thing, was described as important in all situations. However, a challenge with all types of support was asking for help. Participants recognised more active effort from others in offering support was needed, while acknowledging that it may be difficult to notice they are unwell from outside.

“*I have a very supportive network of people around me that I can call on, but I generally don’t. It’s just the nature of the beast I guess […]. So occasionally I’ve spoken to somebody, but normally by the time I’m starting to feel suicidal then it’s—I’ve gone past that point and generally don’t seek help. It’ll be somebody notices something about my demeanour or whatever*.”

Additionally, to be really helpful, peer-to-peer support needs to have qualities of a real, authentic ongoing relationship. This can be challenging to maintain when people work solo in an organisation or in remote areas, where virtual connection to the LEW is the only option. Fostering connections and having sufficiently large peer networks to be able to provide the needed support was described as the means to address this.

“*I think [name] has got like a peer support type thing, where the body just rings up, there’s speakers and that sort of stuff. To me that’s just a very first step. I think you have to meet people. You can’t just do things—I know everything else is done online these days, but, from my perspective, I think it’s incredibly important to have those face-to-face meetings. I thoroughly enjoyed whenever we had a lived experience committee meeting, we would have a meal and coffee or whatever and we’d just have a get together during the day. I made those connections and yeah, that was incredibly important for me, because quite often we feel—well I certainly feel very alone and isolated in that world*.”

### 3.2. Passion

Participants spoke about the intense internal drive to be part of the LEW, which keeps them doing this work. This was described in three ways. Firstly, participants described feeling really motivated when seeing change, knowing that it was possible. Second was the ability to help others, giving a sense of purpose and contributing to feelings of self-worth. Third, they felt that, by doing this work, they could “*create something good out of something bad*”. The LEW helped them make sense of their pain, find purpose in it, or continue to feel the bond with the lost loved one.

“*In my life I’m prepared to share my experience and see whether it can help others. I learnt from the school of life in a couple of ways. So, I see my role to humanity as about definitely doing what I can do to give back—because of this experience—to others*.”

“*For me to feel that I’m honouring a name that it doesn’t just disappear… Keeping it here… Gave me a great… it was very healing for me and it’s very reassuring. It’s like… it’s a connection that’s… in essence gone but you can keep the threads of that connection*.”

In addition to being a motivator, having this strong internal drive can be related to issues of sustainability in certain situations. The first of these is difficulties finding opportunities for work. Participants described feeling highly motivated, but then struggling to find meaningful ways to engage in the LEW and feeling discouraged as a result. Second, participants described difficulties balancing the level of involvement with their own needs, struggling if they needed to step down. Finding the balanced level of involvement seemed to be one of the challenges of continuous involvement related to passion.

“*I find it really overwhelming doing this kind of stuff. I also find sometimes I can neglect my self-care because I almost feel sometimes like an obligation to be involved if that makes sense?*”

Thirdly, they sometimes struggled with feeling undervalued, particularly when they experienced being involved only to meet formal requirements. Similarly, they could feel devastated by slow or no change. Some participants noted that the skill to manage such disappointments develops over time while doing this work, making continued participation easier.

“*I mean I got incredibly upset when the World Health Organisation put out its—I can’t remember, manifesto or whatever—about 4 or 5 years ago. At the launch of their paper about suicide and preventing suicide, there was lots of talk about including lived experience, but there wasn’t one word in the document about it… about encouraging and engaging with people with lived experience*.”

Lastly, some voice fear of doing harm to others through their lived experience work, recognising the different ways their stories can be heard and affect others. Even though this worry can be difficult to bear, it also seems to bring a higher level of reflexivity about the impact of one’s work.

“*That was my biggest fear, that for all the people that I helped that used to come up afterwards and say thank you or—and some people would come up and say, you’ve given me the courage to do something, I was—always had a dread that there were people walking out of that room going, I can’t be here and I can’t do that, I’ve got to—this is—I’m hopeless at this, and that’s the other side*.”

### 3.3. Personal Impact of LEW

Participants admitted that the LEW directly and personally affected them, and, if not mitigated by constant self-care, this could impact their decision to continue the work. There are two sides to the effects of this work; it is intensely emotionally challenging and draining, and it requires courage in showing vulnerability. Participants felt they could not be completely impartial and compartmentalise their work from their life; the need to disclose their own personal story could be draining. Participants also stated that they constantly needed to balance authenticity and disclosure while maintaining boundaries in order to not overwhelm the other person. Participants also described that they could feel emotionally vulnerable when exposing themselves when participating in the LEW; they could hear insensitive things about their own experience or fear that they could break down when doing this work. They also felt that the LEW became an important part of their identity, and this could be painful if that part of their identity was not accepted in their personal space.

“*I would ring and I would get the full story verbally over the phone from the individuals. So, I was struggling. I got—it got to a point where I had to, for my own self-care, walk away from it because I… had overloaded in what I was taking on board and my responses and my management wasn’t as effective as it should have been […]. And it was the vicarious… The vicarious trauma that was going as well…*”

“*The difficulty that it—or it’s not so much the difficulty, but the—what you have to go through to—the courage that you have to have to speak about your own lived experience of suicide as an attempt survivor. It is different I think for somebody bereaved by suicide, or somebody who is caring for somebody who is suicidal. But for those of us who have attempted to take our own lives, yeah, it takes a lot to talk about it*.”

Participants acknowledge that this personal impact of a LEW requires constant self-care. This requires *self-awareness and self-respect regarding self-care needs*. They need to monitor their needs and acknowledge them, acknowledge their own mental health issues, and find help when needed. This also includes knowing their own boundaries in terms of being part of the LEW (how much, when to stop, and when to say no). Such self-reflection, participants suggest, should be fostered within the LEW.

“*I really think it is so important that people are able to be self-aware and self-recognise where they’re at on a day-to-day basis, on a week-to-week basis, look on an hour-to-hour basis. Because especially if you are dealing with people—if you as someone with lived experience are dealing with people who are currently living in that experience at that time that you’re dealing with them, it can be quite a triggering thing for some people. I think it’s so important for them to realise within themselves when it’s time to be able to—to take a step back from that and feeling comfortable and confident to say, hey I need to take a step back from this*.”

Secondly, participants described that an important part of self-care was finding enjoyment and pleasure in their role. The element of enjoyment was specifically highlighted for volunteer positions, because then a person was not getting paid for the work they did. Lastly, participants described personal strategies that helped them destress, such as hobbies, pets, sports, and music.

“*Once you have your core understanding of yourself, you’re doing something that you feel is of value, that you feel you add value to, the people you’re engaging with are relating to you in a mutually respectful and congenial way, the pleasure associated with doing that, is, as I say, a side benefit*.”

### 3.4. Training for LEW

Participants highlighted the need for training for the LEW for it to be sustainable. There were four groups of specific skills that were highlighted as requiring training: (1) *personal wellbeing*, such as self-care skills, knowledge of own strengths, self-knowledge on how work in the LEW can affect them; (2) *helping others*, specifically how to support peers, and how to respond to help others in suicide crisis; (3) *systems knowledge*, such as language and terms used, how to best navigate the system, and how to manage issues arising in reality when doing the work within the existing system; (4) *disclosing*, specifically telling their story related to suicide in public and revealing it directly during peer support.

“*It [training] was giving me the languages that should—could be and must be used in helping people. I’ve done that quite a lot when people talk about taking—committing suicide and… in particular—how important it was to not to see this as a commitment. It means almost like—committing is like a crime*.”

Participants also highlighted that the training needed to be regular and ongoing, be LEW-specific, be of good quality, enjoyable, and accessible, and require workplace support in terms of funding the training and providing information about it. Lastly, if training was insufficient, participants described challenges that occurred.

“*I believe that I went to TAFE with a lot of people who were not ready to be peer workers, and most people gained the qualification by the end of it. The thing that I was super concerned about, and I think it probably just grew after I entered the workforce was if—I don’t know whether these people got work or not—a peer worker, the one thing that they really, really, really need to be educated about is how to disclose difficult things. I think that’s a massive skill, and you can definitely work on it, but if someone’s at a point as a peer worker where they’re getting more therapy out of the relationship than the person, that is a huge concern to me*.”

### 3.5. Diversity in LEW

Participants talked about diversity within the LEW in terms of the diverse roles they had, the different types of experiences they brought, and differences from other types of workforces, such as mental health LEW and non-LEW in suicide prevention. How successfully these diversity issues are navigated links back to the sustainability of the workforce.

LEW contains multiple different roles, and participants commented on the need of self-awareness with regard to the range of activities one can most successfully do in the workforce.

“*I think a diversity of input, for a start. Be that training, be that opportunity and experiences. Different organisations, connecting with different organisations. Basically, broadening the types of information that’s coming in to you, so that you can sort of start to build a clear picture of what the space is, what it requires, how you can best be engaged with it, and where you think you can provide the most benefit. In other words, what your strengths are*.”

Some participants saw lack of diversity in the workforce as an issue, in terms of imbalance of the experiences reflected within the LEW. Seeing their voice underrepresented in terms of type of lived experience, or their more complex story not fully appreciated, could feel as if it was being undervalued and, thus, could create disappointment.

“*And another problem I’m seeing is I’m noticing more and more that when I am in various spaces with people with the lived experience of suicide, those spaces are starting to be quite dominated by people who’ve been bereaved by suicide rather than people who have survived a suicide attempt*”

Participants also spoke about the difficulties demarcating the suicide prevention LEW with regard to mental health lived experience and the non-LEW in suicide prevention. They saw both overlap and differences with the issues they faced, and both drawing too much distinction and identifying them as completely equal could create difficulties with sustainability of the workforce.

“*Just because they don’t identify as someone with a lived experience, they don’t stand up with a bloody flag, waving it, like I have to do, it doesn’t mean that they haven’t been affected by these things, and I almost felt that they missed out because they don’t have the title peer worker*.”

“*Talking about staff attitudes, an attitude that’s coming through quite a bit—so when you do codesign stuff where you might have clinicians and lived experience or whatever and the clinicians will often say, I have lived experience too. It almost feels like they’re saying, so we don’t need you because I’m a clinician and I have lived experience so I’m a whole-package deal, whereas you’re not a clinician and you have lived experience, so you’re irrelevant*.”

## 4. Discussion

Despite a general increase in research on lived experience in suicide prevention, there is still limited information about the suicide prevention LEW and its sustainability. The aim of this qualitative study was to further explore the sustainability of the suicide prevention LEW. We identified five main themes: support, passion, personal impact of LEW, training, and diversity within the LEW.

The need for support and regular training within the suicide LEW seems to mirror findings of factors sustaining mental health LEW [6,38]. This study highlights that support is needed from both within the system that employs the person, as well as a wide range of ongoing training needs. Such support and training could be incorporated with the expansion of the LEW within the national mental health and suicide prevention sectors. Training for professional development and career pathways for those with lived experience has been suggested more broadly, as well as training for managers and supervisors [6]. However, training related to self-care in terms of suicidality, as well as disclosing one’s own suicidality, seems to be specific to suicide prevention LEW. This form of training has not been mentioned in training of mental health LEW [6,39]; therefore, such training is needed specifically for the suicide prevention LEW.

The descriptions of the passion theme indicate the strong internal drive with which these individuals undertake their work. This may be a positive in terms of the vigour needed to undertake the complex tasks required of them within the suicide prevention LEW which, as participants described, is still frequently based on voluntary and unpaid involvement. However, there seems an inherent tension between passion and the personal impact of participation in the LEW theme. There is an ongoing difficulty, inevitably embedded within the LEW, in finding the balanced level of involvement while meeting the needs for deriving meaning from this work, but not overburdening the individual. This constant tension between passion for the work and personal impact of LEW involvement was described as requiring ongoing self-awareness and self-respect regarding self-care needs and is also similar to the self-care mechanisms deemed essential for the mental health LEW [6]. Built-in mechanisms to recognise this unavoidable tension and foster self-reflection for the participants of the LEW and those employing them could be helpful.

Diversity within the LEW in terms of roles and types of lived experience seem to bring both challenges and opportunities for the workforce. The selection of the precise type of work one wants to do from the variety of roles, which are no longer limited to peer support or telling one’s own story, seems to require self-reflection, appropriate training, and access to these different opportunities of work, since not finding such a role seems to lead to lower involvement. Similarly, seeing one’s unique lived experience as undervalued within the workforce can bring similar doubts about involvement. Respecting the full scale of diversity of lived experience that people bring has been highlighted as a challenge within the mental health LEW [6,12], and this issue seems to arise within the suicide prevention LEW workforce. The findings suggest that this seems to be an ongoing issue, allowing for a variety of lived experience voices, while ensuring that no single narrative or only some narratives are permitted to dominate.

The issues surrounding specificity of LEW are linked with the issues of identity and role clarity in relation to peer support that have been raised in the mental health sector. In a study by King and colleagues [40], findings reported that identity management and the added “cognitive load” of managing a lived experience whilst working in designated lived experience roles created additional challenges for peer workers. For example, Huisman and van Bergen [22] highlighted that clarity is needed around the role of suicide prevention peer workers, and that this should be discussed between them and clinicians. Further, Roennfeldt and Byrne [41] referred to the professionalisation of the mental health LEW, highlighting concerns around professional identity and credibility of those with lived experience as viewed from within the system. In particular, they advocated for the role of lived experience to be seen as a distinct professional discipline. The findings of the present study suggest that these questions are relevant for sustainability and feelings of being valued in their roles within the suicide prevention workforce, but the participants were less clear whether this distinction would be helpful or limiting. The issue of identity of lived experience, personal impacts of this lived experience, and whether there is a need for defining designated lived experience roles as a requirement to participate in this workforce has also been raised in other studies [20,41,42]. Open discussions about the distinctions and overlaps between LEW and non-LEW within the suicide prevention sector seem to be needed, since there is no clear and uniform answer in this case.

## 5. Limitations

There are several limitations of the study that are noteworthy. Recruitment focused on those completing the training specific to the suicide prevention LEW. It is possible that the sample reflects only part of the LEW which has had more support and training, whereas those working without having received such suicide-specific training may face different issues and offer additional perspectives. Throughout the coding and analysis process, we attempted to have a balance of researchers with and without experience within the suicide prevention LEW to be able to perceive the participants’ narratives from different viewpoints and integrate these into the analysis. However, we acknowledge that the experience within the LEW by some of the research team, as well as the recruitment process could impact the analysis by focusing the themes and their labelling around the narratives held by those who have been longer in the field rather than those who leave the LEW quickly after engaging in it. Wider discussions during the development of the guidelines for sustained participation of the suicide prevention LEW should address this gap. In addition, a wider sample of those working in suicide prevention LEW is still required to test the relative importance of each of the identified themes, as well as find the best mechanisms to implement them.

## 6. Conclusions

The main themes identified in this study can be used in creating measures to facilitate sustained participation in the suicide prevention LEW. Prior studies have recommended considerations for responding to potential pitfalls and further action be taken to elucidate the role for ongoing workforce participation difficulties [6,22]. The results of this study suggest managing the expectations of LEW participants by providing clear information about the roles and nature of the job, the courage required, career pathways within the LEW, and how stepping-down can impact the workers is important. It is of equal relevance that the LEW is given the tools to both manage and navigate the question of meaningful involvement without overburdening themselves. This knowledge can be translated into participation and support guidelines for the suicide prevention LEW to support sustainability.

## Figures and Tables

**Table 1:** Participants involvement in different types of LEW activities.

Types of Activities Involved in	Participant’s Number												
	1	2	3	4	5	6	7	8	9	10	11	12	13
Codesign of programs/services	•	•	•		•			•	•	•	•	•	•
Co-evaluation of programs/services/research		•		•	•					•			•
Participated in a lived experience reference, working, or advisory group	•	•	•	•	•			•	•	•	•	•	•
Participated in strategic planning for a suicide prevention-related organisation	•	•	•					•		•	•		•
Involved in governance activities	•	•	•		•				•				
Employed as a peer worker		•							•				•
Helped deliver or facilitate a lived experience or peer-led service/program		•							•			•	•
Delivered lived experience-led training to staff		•				•		•					•
Lived experience speaker	•	•	•		•	•	•	•		•			•
General volunteer positions		•	•		•								
Attended a community consultation about suicide prevention		•	•		•			•		•			•

## Data Availability

The data presented in this study are available upon reasonable request from the corresponding author.
